# Post-Traumatic Orbital Reconstruction Using Titanium Patient-Specific Implants: A Clinical and Radiological Cohort Study Focusing on Paranasal Sinuses Physiology

**DOI:** 10.3390/jcm14207439

**Published:** 2025-10-21

**Authors:** Waldemar Reich, Louis Widmaier, Ulrich Kisser, Jens Heichel, Sven Otto, Frank Tavassol

**Affiliations:** 1 University Hospital for Oral and Plastic Maxillofacial Surgery, Martin Luther University Halle-Wittenberg, Ernst-Grube Str. 40, 06120 Halle (Saale), Germany; 2 University Hospital for Otorhinolaryngology, Head and Neck Surgery, Martin Luther University Halle-Wittenberg, Ernst-Grube Str. 40, 06120 Halle (Saale), Germany; 3 University Hospital for Ophthalmology, Martin Luther University Halle-Wittenberg, Ernst-Grube Str. 40, 06120 Halle (Saale), Germany; 4 University Hospital for Oral and Plastic Maxillofacial Surgery, Head of Department, Ludwig Maximillians University Munich, Lindwurmstraße 2a, 80337 Munich, Germany; 5 University Hospital for Oral and Plastic Maxillofacial Surgery, Head of Department, Martin Luther University Halle-Wittenberg, Ernst-Grube Str. 40, 06120 Halle (Saale), Germany

**Keywords:** trauma, orbita, patient specific implant, paranasal sinus, titanium, adult, midfacial fracture, osseointegration, Lund score, reconstructive surgery

## Abstract

**Background:** This longitudinal cohort study evaluated implant-associated bone remodeling and paranasal sinus (PNS) status after the insertion of patient-specific titanium orbital implants (PSIs) in adult trauma patients. Sixteen patients with various orbital fractures underwent CT-based reconstruction at the University Hospital Halle (Germany) and were followed up to 6.5 years (observation period February/2019–October/2025). Post-operative CT scans assessed orbital bone remodeling, patency of the ostiomeatal unit, and PSI/screw exposure. **Findings**: Bone apposition was observed in 16 cases; 13 showed a patent maxillary sinus outflow tract. The median Lund score for the injured sides was 1.0 vs. for the uninjured sides 0 (Wilcoxon test, *p* = 0.131). PSI or screw exposure occurred in isolated cases, and basal maxillary sinusitis was noted in four patients. Significant bone remodeling was detectable from 6 months post-operatively. No implant-associated complications required further intervention. **Conclusions and Relevance**: These findings highlight the safety and precision of PSIs, with low long-term complication rates and preserved sinus function in non-irradiated patients, supporting their use in complex orbital reconstructions.

## 1. Introduction

### 1.1. Virtual Surgical Planning and CAD/CAM Technologies in Craniomaxillofacial Surgery

Three-dimensional technologies (computer-aided design/computer-aided manufacturing [CAD/CAM]) have generally become more attractive and widespread in reconstructive surgery in recent years. They help to improve the accuracy and predictability of bone reconstructions and shorten the duration of surgery [1].

**Figure 1:**
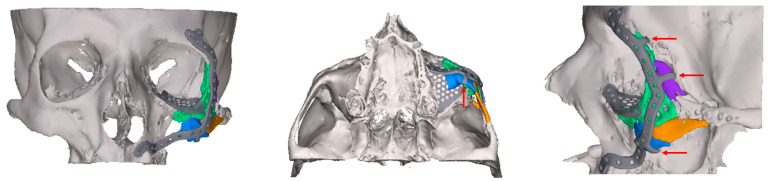
Virtually planned 3D-reconstruction of complex lateral midface trauma (example, titanium plate with a thickness of 1 mm, orbital implant with a thickness of 0.3 mm). Colored fragments of the zygomatic bone were virtually repositioned and stabilized by means of 3 flaps (red arrows).

Patient-specific alloplastic implants are used for the primary and secondary reconstruction of complex craniofacial defects (Figure 1), including orbital wall fractures [2,3,4]. Orbital implants are made of titanium [5] and plastics such as polyether-ether-ketone [6] or resorbable magnesium-based [7] or bioactive composite materials [8].

Based on the preoperative diagnostic computed tomography (CT) data sets in DICOM format, 3D technology is routinely used in alloplastic reconstruction in adults as well as in the rehabilitation of pediatric patients, e.g., with craniofacial synostoses [9]. This new generated information for 3D printing is based on the standard tessellation language (STL) format. In basic research, regeneration is also possible using living biological matrices or biological matrices colonized with cells (scaffolds) [10,11].

### 1.2. Research Background and Study Objective

**Figure 2:**
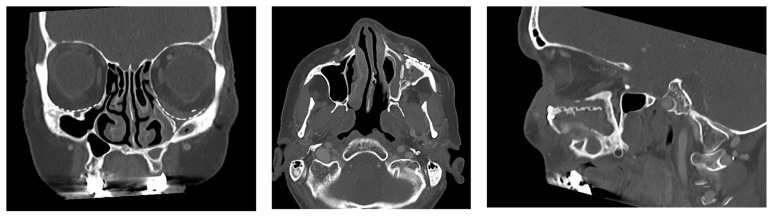
Late complication 15.5 years after a bilateral centrolateral midfacial fracture in an adult woman. Displacement/mispositioning of the classic orbital mesh on the left side, acutely exacerbated maxillary sinusitis, an obstructed drainage pathway requiring exploration (mesh removal) and infundibulotomy.

Most clinical studies in the field of post-traumatic reconstruction of the inferior and medial orbital walls focus on accuracy of implant placement and implant surface contouring. The intraorbital soft tissue [12] as well as the paranasal sinuses require consideration. It is known that sinogenic complications may affect the reconstructed orbita [13]. The anatomy and physiology of paranasal sinuses are extensively investigated [14]. Although the maxillary sinus is different from the frontal and ethmoid sinuses, there is a potential risk of developing maxillary sinus mucocele as a late complication in midfacial complex fractures [15] as well as sinusitis (Figure 2). According to an earlier questionnaire-based study of 34 patients (SNOT-20, a validated, disease-specific, health-related quality-of-life survey for chronic rhinosinusitis), the authors concluded that patients who suffered from midface fractures have a much higher risk of developing chronic rhinosinusitis [16].

Reconstruction of orbital defects remains a major challenge in craniofacial surgery, not only because of the complex anatomy, thin bony walls, and individually variable shape of the orbit but also due to its close relationship to the paranasal sinuses. While advances in computer-assisted planning and patient-specific implants have improved accuracy in orbital wall reconstruction, post-operative complications such as sinus dysfunction may impair long-term outcomes. Disruption of normal sinus drainage or aeration after orbital reconstruction may contribute to chronic sinusitis, mucoceles, or impaired orbital healing. A deeper understanding of how reconstructive techniques influence sinus physiology is therefore essential.

**Figure 3:**
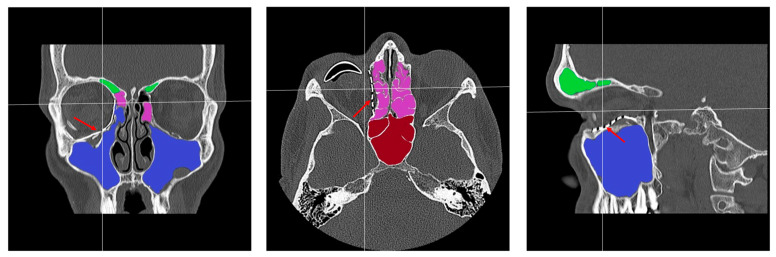
Post-operative CT (example) with colored PNS (green—frontal, violet—anterior and posterior ethmoid, magenta—sphenoid, blue—maxillary sinuses) and PSI in situ (red arrows).

The primary objective of the present study was to investigate the clinical and radiological results of reconstruction with individual patient-specific implants (PSIs) in patients with post-traumatic complex orbital defects with regard to the positional relationship to the paranasal sinuses (PNS, Figure 1, Figure 2 and Figure 3). Midface fractures almost always result in hemosinus, and blow-out fractures are characterized by structural narrowing of the outflow tract of the maxillary sinus. To our knowledge, there are no studies in the literature on functional disorders of the PNSs following orbital reconstruction with alloplastic PSIs. This extended analysis follows on from our previous study on the reconstruction of orbital floor fractures with pre-formed titanium meshes [17].

We hypothesized that orbital reconstruction using PSI does not cause chronic sinusitis due to the following reasons: 1. It maintains the patency of the maxillary sinus drainage (ostiomeatal complex). 2. It ensures sufficient bone remodeling to the adjacent, traumatized paranasal sinuses, and 3. It provides a reliable implant success rate.

## 2. Materials and Methods

### 2.1. Study Participants

Trauma patients with complex orbital defects who underwent surgical treatment from February 2019 to May 2024 (recruitment period) at the Department of Oral, Maxillofacial and Plastic Surgery of the University Hospital Halle (Germany) were analyzed. The inclusion and exclusion criteria are listed below. Surgical treatment was basically indicated if one or more of the following criteria were met:

- Clinical criteria [18,19]
  ○ Enophthalmos greater than 2 mm
  ○ Diplopia
  ○ Entrapment of the inferior rectus muscle
- Radiological criteria [20]
  ○ Defect size ≥ 1 cm^2^ or defect over 50% of the orbital floor
  ○ Herniation or incarceration of orbital contents, in particular degree of dislocation of the inferior rectus muscle.

The study participants were prospectively and consecutively included according to the following inclusion and exclusion criteria. Regarding the timing of surgery, early reconstruction—within 2 weeks after trauma—was favored [21].

Inclusion criteria:

- Adult patients aged ≥ 18 years
- 3D bony defect in the orbital region
- Complex unilateral orbital fractures—category ≥ II according to Jaquiery [22]
- Indication for a PSI as part of the primary or secondary reconstruction based on the defect size according to Dubois [23].
- Availability of the follow-up examination data.

Exclusion criteria:

- Previous midface trauma
- Tumor patients
- Cystic fibrosis
- History of radiotherapy in the midface area
- Trauma patients in whom osteosynthesis can be performed exclusively with ready-made material
- Category I orbital floor fractures according to Jaquiery [22].

The primary outcome measure of this study was the CT-based Lund numerical score [24] in relation to the PSI (see below). This established score is used for quantitative radiological assessment in patients with chronic sinusitis.

### 2.2. Digital Workflow and Implants

**Figure 4:**
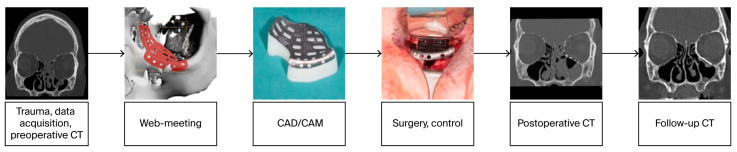
Digital workflow.

Figure 4 shows the treatment pathway from data acquisition to post-operative follow-up. Virtual surgical planning and implant manufacturing (selective laser melting—SLM) were carried out in cooperation with an industrial partner (KLS Martin Group, Tuttlingen, Germany) as previously described [4,25]. The software for the CT data management and 3D design was Mimics medical^®^, version V27.0.0.527, Materialise, Leuven, Belgium und Freeform^®^, version 2024.0.87, 3D Systems, Rock Hill, SC, USA. The preferred surgical accesses were the transconjunctival and mediopalpebral/subtarsal approaches. In all cases, the orbital implants were inserted using a small anatomical clamp without intraoperative navigation. It was ensured that the PSI (implant thickness of 0.3 mm) was positioned as accurately as possible on intact bone edges anteriorly, medially, and laterally. All implants were precisely fixed on the infraorbital rim with at least two titanium microscrews (4–5 mm length), and the implant position was then clinically verified again as described above. All patients received intraoperatively 250 mg methylprednisolone and an antibiotic prophylaxis with ampicillin/sulbactam for 3 days. Planning and reconstruction were carried out by the first author of this article.

### 2.3. Data Collection and Defect Morphology

The following pseudonymized parameters were recorded in a table: age, gender, diagnosis, therapy, defect morphologies of the orbital fractures according to Jaquiery [22] based on the CT data, type of PSI, surgical access, duration of surgery, intraoperative peculiarities in handling the PSI, any complications, their treatment, eyeball mobility, and follow-up period (Table 1). Complex fracture patterns exceeding the categories according to Jaquiery [22] were marked by “+”.

To consider potential influence of coexisting diseases as confounding factors in rhinosinusitis pathophysiology, the records also included the following: a history of nasal and/or sinus surgery, allergic rhinosinusitis, chronic airway diseases [27].

All patients underwent preoperative and post-operative ophthalmologic examinations (visual acuity, tension, motility, diplopia, cornea, retina). Ophthalmologic treatment results (Table 2) during the follow-up were classified according to Jansen [28]. In addition, patients were explicitly asked about any symptoms of acute sinusitis and were clinically examined for these symptoms.

The radiological assessment of the orbit and the paranasal sinuses after orbital reconstruction of varying complexity focused on the key regions in three planes: posteromedial bulge (axial), transition zone (coronal), post entry zone, infraorbital rim and recessus (sagittal), posterior ledge (coronal, sagittal).

**Figure 5:**
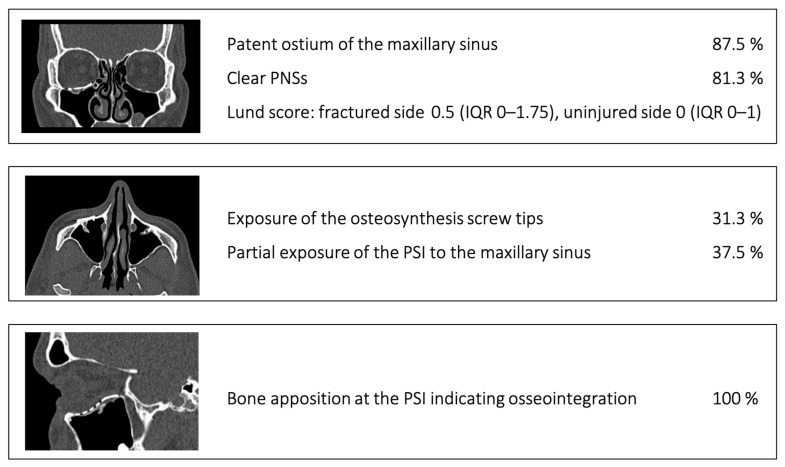
Main radiological findings of the CT examination after orbital reconstruction using PSI.

The following five aspects were assessed during the radiological follow-up using CT (bone window, Figure 5):

- Patent ostium of the maxillary sinus (coronal plane)
- Non-inflammatory PNSs (three planes)
- Exposure of the osteosynthesis screws (axial and sagittal planes)
- Exposure of the PSI to the maxillary sinus (coronal and sagittal planes)
- Remodeling of the fractured orbital walls and bone apposition at the PSI indicating osseointegration (three planes)

The radiological assessment of the PNSs (last follow-up CT) was performed by the first author as follows using the Lund numerical score for both sides [24,29]: assessment of pathological mucosal swelling (0: no abnormalities, 1: partial opacification, 2: total opacification) in the maxillary sinus, in the anterior and posterior ethmoid cells, in the sphenoid sinus, in the frontal sinus, and in the ostiomeatal complex (0: not occluded, 2: occluded). In patients who required frontobasal coverage with frontal sinus cranialization, the score for this side was assigned as “0”. The maximum score per side is thus 12 points. Due to hemosinus in the initial CT scans, a reasonable assessment of the Lund score at the time of recruitment and postoperatively was not possible.

As part of the qualitative analysis of remodeling, the three planes (bone and soft tissue windows) of the (a) preoperative CT, (b) post-operative CT, and (c) follow-up CT were compared with each other. Therefore, up to 18 data sets were available per patient. We scrolled and looked through all CT scans available in every direction (axial, sagittal, coronal). The unoperated orbita side served as a control. For this study, native CT scans (Siemens healthcare, SOMATOM X.cite CT scanner, Forchheim, Germany) of the skull from the Department of Radiology of the University Hospital Halle (Germany) or from the referring clinics and colleagues were used.

### 2.4. Consent, Data Management

The study has been approved by the Ethics Committee of the Medical Faculty of Martin Luther University Halle-Wittenberg (processing number: 2018-131). The participants provided informed consent to take part in the study.

After an initial intensive post-traumatic follow-up, a routine annual clinical examination was planned and carried out in the patients analyzed here.

This manuscript was prepared in accordance with the STROBE (Strengthening the Reporting of Observational studies in Epidemiology) statement checklist.

### 2.5. Statistics

The statistical analyses were carried out using IBM SPSS statistics (Version 28 and lastly 31.0.0.0, Chicago, IL, USA). Absolute and relative frequencies as well as combinations of characteristics were analyzed descriptively. To compare the mean ranks of the Lund scores, the non-parametric Wilcoxon test for paired and unpared samples and Pearson’s correlation were performed at a significance level of *p* < 0.05.

Based on the prevalence of chronic rhinosinusitis of 10.9% per year in Europe [30] and the mean Lund score of 4.26 [31], the calculated sample size was at least 66 orbitae. The statistical power was set at 80%. In a “split-face” design (contralateral side-control) we aimed to include 33 patients. In the present manuscript we would like to present the first results.

## 3. Results

In the present study, 16 (9 male, 7 female) of 35 patients met the inclusion criteria in the recruitment period. The last patient of the presented cohort study was recruited in May 2024. In 19 patients, a CT scan was missing in the post-operative follow-up interval as patients did not return for follow-up investigation (dropouts). The overall observation period was more than 6.5 years (February 2019–October 2025).

The patients included had the following fractures (Jaquiery category II–V):

- Fracture of the orbital floor (*n* = 3)
- Fracture of the orbital floor + medial orbital wall/zygomatic bone (*n* = 7)
- Complex centrolateral midface fracture, in some cases with involvement of the skull base (*n* = 6).

The bone reconstruction required the insertion of 20 implants: one implant *n* = 13 (orbital floor *n* = 7, orbital floor and medial orbital wall *n* = 6), two implants *n* = 2 (orbital floor and medial orbital wall), three implants *n* = 1 (orbital floor and medial orbital wall, zygomatic bone, zygomatic arch). The mean duration of surgery was 175 min (range: 60–360 min). In addition to primary soft tissue closure, two cases required a canthopexy, and another patient had a paranasal soft tissue deficit. The details are summarized in Table 3.

The classification of treatment results according to Jansen [28] revealed an excellent result in 13 cases and an acceptable result (diplopia during extreme upward gaze) in 1 case. Due to the primary eyeball injury, amaurosis occurred in one case (patient 12, Table 3) despite ophthalmic surgery, while another case (patient 15, Table 3) had reduced visual acuity of 0.25 (corneal scar, traumatic cataract).

In addition, the clinical long-term follow-up revealed occasional serous rhinorrhea in two cases, and hypo-/hyper- or paresthesia in the area supplied by the infraorbital nerve in five cases. The recall examination was mostly performed in the autumn and winter, when patients are susceptible to seasonal rhinosinusitis. The participants did not complain about allergic rhinosinusitis.

The median Lund scores were distributed as follows: for the fractured side 1.0 (IQR 0–2.75), range 0–6, for the uninjured side 0 (IQR 0–1.75), range 0–5. Both scores correlated with each other (Pearson’s correlation *r* = 0.9, *p* < 0.001). The median Lund score for the frontal, posterior ethmoid, sphenoid sinuses, and the ostiomeatal complexes on the injured sides was 0 (IQR 0–0). The median Lund score for the maxillary and the anterior ethmoid sinuses on the injured sides was 0 (IQR 0–1). The median Lund score for the frontal, anterior and posterior ethmoid, sphenoid sinuses, and the ostiomeatal complexes on the uninjured sides was 0 (IQR 0–0). The median Lund score for the maxillary sinuses on the uninjured sides was 0 (IQR 0–1). There were no significant differences in the median values depending on the injured vs. uninjured sides (Wilcoxon test for paired samples, *p* = 0.131) as well as the complexity/category of the orbital fracture according to Jaquiery (Wilcoxon test for unpaired samples; *p* = 0.736). Three patients required frontobasal coverage with frontal sinus cranialization and scoring for this side was assigned as “0”. Opacity of the frontal sinus was observed only in one case and the ostiomeatal complex was obstructed in two cases. In two other cases, the maxillary sinuses were reduced in volume while totally transparent (Table 4). There was no evidence for a maxillary sinus mucocele.

As potential confounding factors, there were the following: a history of an allergic rhinoconjuncitivitis in three cases, and a single case of septoplasty, as well as Churg-Strauss syndrome.

The details of the clinical and radiological follow-up examinations of the individual patients are presented in Table 4. All orbital PSIs remained in situ. There was no evidence of sino-cutaneous fistula or implant exposure at the infraorbital rim. The current cohort presents an interim analysis.

The follow-up CT scan was performed after an average of 19 months (range: 6–28 months). The radiological findings were as follows (Figure 5):

- Patent outflow tract of the maxillary sinus (*n* = 14) and physiologically ventilated PNSs (maxillary sinus, ethmoid cells, *n* = 13)
- Exposure of the fixation screws (infraorbital recess of the maxillary sinus) and limited exposure of the PSI (transition zone, ethmoid cells) without signs of mucosal swelling in these regions (*n* = 6 each)
- (Basal) mucosal swelling in the maxillary sinus indicative of chronic maxillary sinusitis, irrespective of the complexity of the primary injury (*n* = 4)
- Bone apposition at the PSI as a sign of osseointegration (*n* = 16)
- Remodeling of the dislocated bone fragments of the orbital walls (*n* = 16), including in the area of the infraorbital canal.

**Figure 6:**
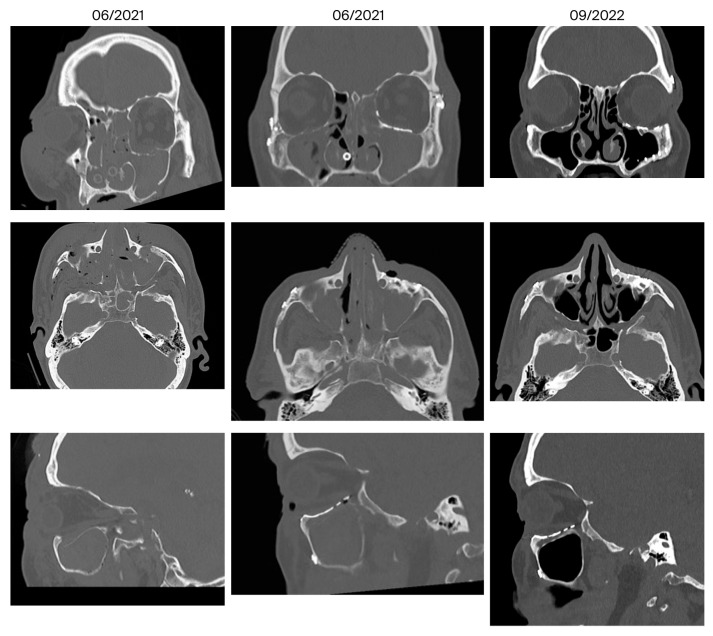
Reconstruction of a centrolateral midface fracture (patient 13 in Table 3, category III+ according to Jaquiery [22]). Lund score 0/1 (right/left).

**Figure 7:**
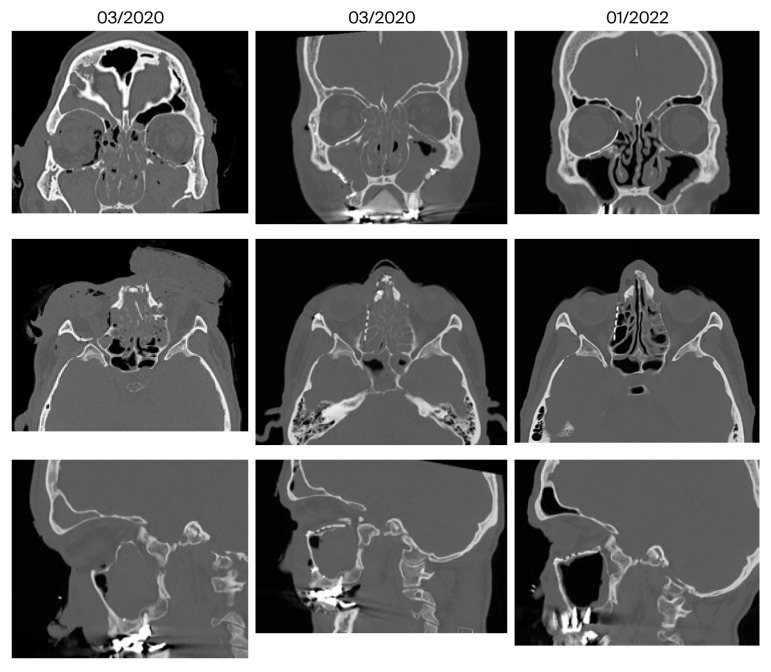
Reconstruction of a centrolateral midface fracture (patient 10 in Table 3, category IV+ according to Jaquiery [22]). Lund score 3/5 (right/left).

**Figure 8:**
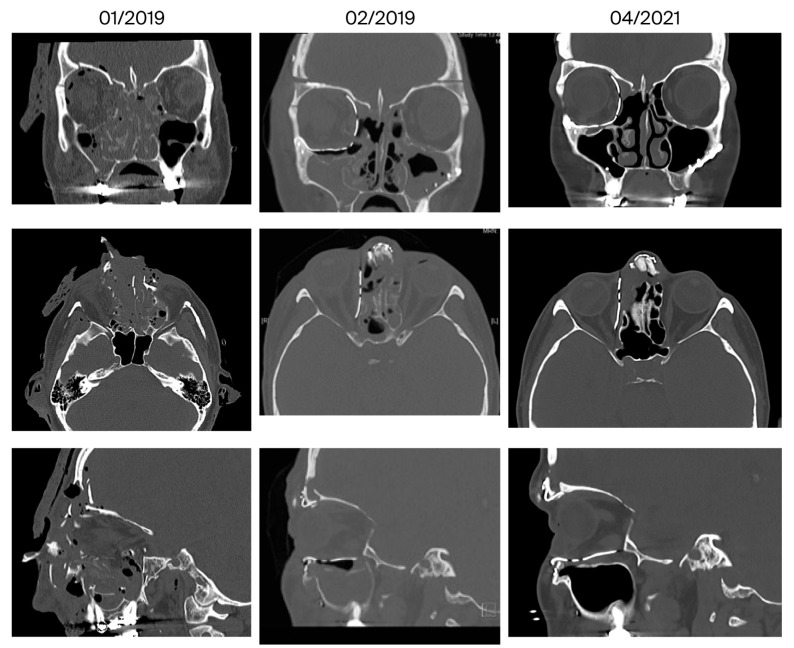
Reconstruction of a centrolateral midface fracture involving the anterior skull base (patient 9 in Table 3, category V+ according to Jaquiery [22]). Lund score 5/5 (right/left).

Figure 6, Figure 7 and Figure 8 show representative CT images (bone window) for different and in-creasing degrees of trauma severity at the three examination time points. The coronal plane of the CT examination of patient 13 in Table 3 is presented as Video S1 to give the reader a comprehensive overview.

## 4. Discussion

### 4.1. Orbital Floor Fractures and Complex Centrolateral Midface Fractures

The possibility of an exact reconstruction of the complex orbital anatomy in large single-wall and multi-wall fractures as well as easier positioning with limited intraoperative visibility are known advantages of a PSI [4,25,32]. This results in a shorter operating time and a reduction in intraoperative trauma to the orbital soft tissues due to mechanical manipulation and therefore less post-operative swelling [33]. In a recent single-center study in which the implants were positioned with the freehand method without intraoperative navigation, the authors compared the preoperative virtual planning and the CT-based post-operative position of the implants and found a median difference of 0.39 mm in the mean surface distance at the orbital floor and 0.42 mm at the medial orbital wall [34]. Based on a prospective multicenter study, including 195 patients, it was shown that CAD-based individualized orbital implants provided the most precise reconstruction in terms of post-reconstruction orbital volume compared to pre-formed meshes. The authors stated additionally that with intraoperative navigation, the precision of orbital volume reconstruction increased significantly. They found that differences in volume between the reconstructed and the unaffected orbit ranged from 3.5 mL to 1.4 mL for non-CAD-based and from 2.0 mL to 0.6 mL for CAD-based individualized implants. The variance of these differences was 1.8 mL^2^ in patients treated with pre-formed and 0.6 mL^2^ in patients treated with individualized implants. However, the variance of the difference between reconstructed and unaffected orbital volume was only 0.7 mL^2^ when navigation was used to control implant shape and it amounted to 1.5 mL^2^ when no navigation was used for shape control. In this study, no statistically significant differences in motility or diplopia were seen between the treatment groups 12 weeks after surgery [33].

Furthermore, based on our experience, by extending the PSI (medially and laterally), a segmental fracture can also be treated infraorbitally by the PSI. In the secondary reconstruction of an anophthalmia or phthisis bulbi, PSIs offer the possibility of an overcorrection with the aim of improving esthetics [35].

The choice of the access route to the orbit is based on the localization of the fracture and, if applicable, the presence of occasional wounds. The use of the proposed “clock model” or “round the clock access to the orbit” is helpful here [36,37]. Due to the limited intraoperative visibility and the need for appropriate access, other authors recommend dividing larger orbital implants into a two-piece puzzle [38,39]. In agreement with other authors, multiple implants were used in our cohort for three- to four-wall defects [40]. Here, special additional design elements (matrix–patrix, puzzle, hooked bar) can facilitate precise interlocking intraoperatively [41]. In Patient 9 (Table 3), the initial one-piece implant had to be replaced with a two-piece implant.

For multi-fragmented centrolateral midface fractures, the additional use of PSIs for the zygomatic bone and, if applicable, the zygomatic arch region enables precise reconstruction. Although PSIs are not superior to conventional miniplate osteosynthesis in this context [42], they offer an intraoperative time advantage, especially in combination with orbital implants. Furthermore, intraoperative navigation and intraoperative 3D imaging represent an improvement in personalized computer-assisted surgery [41].

### 4.2. Paranasal Sinuses

In addition to the known challenges in the regeneration of the orbital floor [43], sparing the PNS mucosa is of great importance in terms of reducing intraoperative trauma and in analogy to functional endoscopic sinus surgery, FESS [44], as the described healing processes after PNS surgery in the acute phase also occur in trauma patients [45]. The present study shows that ethmoidectomy, which is occasionally discussed, is not necessary for comminuted fractures of the central midface.

In addition, while focusing on paranasal sinuses physiology, attention should be paid to minimizing mucosal trauma caused by stents or tampons [46].

In the current literature, reports on the function of paranasal sinuses in orbital trauma patients restored with PSI are still underrepresented. The question of potential inflammatory complications when the implant is exposed to the PNSs has not been adequately investigated. A literature search in the Pubmed database using the keywords “customized” or “patient specific implants” and “sinusitis”, as well as “orbita*” and “Lund score” yielded no hits. In the present study, minor exposure was observed on morphological CTs in 6 of 16 cases (38%). As can be seen in Figure 6, Figure 7 and Figure 8, after reconstruction of the orbital floor with a PSI, the fractured orbital floor undergoes remodeling and the PSI shows a bone apposition to the maxillary sinus, which can be interpreted as an indication of good biocompatibility. In a case series of trauma patients who had received a PEEK implant for midface reconstruction in close proximity to the PNSs, no sinusitis was observed and explantation due to extrusion/skin dehiscence and bacterial infection occurred only sporadically [47].

A comparative study on bacterial biofilm formation on titanium vs. PEEK surfaces showed higher adhesion on PEEK samples [48]. In a systematic review, however, PEEK implants were found to be superior to autologous bone (OR 0.547) and titanium meshes (OR 0.170) with regard to the frequency of material-associated complications and the need for reoperation [49].

The very low median Lund score in the first presented results of the study is surprising in view of an earlier analysis on patients undergoing a CT scan of the paranasal sinus region for non-sinusitis causes. In 91 adult patients, the imaging revealed a Lund score with a mean of 4.26. The authors concluded that a Lund score ranging from 0 to 5 may be considered within “normal” range [31], which reflects exactly the range in the presented cohort.

In a recent retrospective case–control study, the authors aimed to identify risk factors for maxillary sinus pathology after surgery for midfacial fractures [50]. Patients suffering from orbital trauma are not mentioned explicitly. The multivariate analysis indicated that risk factors for maxillary sinus pathology (total 372 maxillary sinuses analyzed) were as follows: number of screws penetrating into the sinus (OR 1.12), imperfect reduction (OR 2.90), and number of sinus walls involved (OR 1.38). This data supports the indication for use of PSI and removal of osteosynthesis material in the maxilla 6 months post-operatively.

Furthermore, a rare but potentially relevant condition is the silent sinus syndrome, an atelectasis of the maxillary sinus, resulting in hypoglobus, enophthalmos, and diplopia. The main pathophysiological starting point is the obstruction of the ostiomeatal complex, leading to hypoventilation and collapse of the maxillary sinus walls [51,52], which should be considered especially in patients diagnosed/treated with orbital floor and medial wall fractures. Our study demonstrated that PSIs promote (being an osteoconductive scaffold) indirectly the patency of the outflow tract. It should be considered that the prevalence of chronic rhinosinusitis per year in Europe is calculated as 10.9%, which means it is a common health problem with an impact on midfacial trauma treatment [30].

### 4.3. Remodeling

The basic process of bone remodeling has been extensively studied [53], with osteocytes playing an impressive major role in mechanotransduction [54,55]. Computer-assisted models help to describe the highly complex process on multiple scales and apply it to implant osseointegration [56]. The healing processes in midface fractures, and here specifically in the orbit, should also be viewed against this background. Bone remodeling of the orbit occurs spontaneously throughout life [28], depending on age [57], in orbital space-occupying conditions such as endocrine orbitopathy [58], and in benign periorbital space-occupying conditions [59].

In a recent study of 372 CT scans, a perfect orbital symmetry was confirmed, with a tendency to increase with age. These results allow the mirroring of the healthy orbit for established surgical planning [60]. This is also applicable to oncologic patients, as the developmental progress in orbital reconstruction, which is still in progress, has become a driving force in other areas of reconstruction [61].

In the present study, almost all cases of orbital floor reconstruction did not result in complete reduction in the prolapsed bone fragments, which was not necessarily the aim due to the complexity of the procedure. Nevertheless, impressive remodeling occurred during the wound healing phase along the biocompatible implant surface as a scaffold, on the orbital floor, and on the medial orbital wall, even in the case of pronounced dislocation of the fragments. Thus, as evidenced by morphological CT, there is bone contact between the PSI and the remodeled orbital wall that was not present in the immediate post-operative period. Against this background, we believe that the insertion of antral balloons to stabilize the orbital floor via an additional transnasal route [62] is superfluous and unnecessarily invasive. Instead, resorbable radio-opaque PSIs will reach the stage of clinical routine in the future [7,63].

By modifying the manufacturing process (SLM) it is possible to further improve wettability, biocompatibility, and osseointegration of PSI [64] as well as antibacterial properties [65].

## 5. Conclusions

The present study demonstrates that the applied titanium PSIs are reliable and safe tools to restore orbital walls and ocular function in adult patients who suffered from extensive isolated or multi-wall orbital fractures. Regarding the adjacent PNSs, there were no significant differences in the median Lund scores of the injured compared to the uninjured orbita sides. This finding supports the hypothesis that the physiology of the neighboring PNSs is maintained in the long term. Bone remodeling consistent with osseointegration was observed radiologically as early as 6 months post-operatively. As the orbital PSIs behave as an osteoconductive scaffold, it is favorable to intraoperatively preserve sinus mucosa and bony fragments, which are attached to the mucoperiosteum. Exposure of fixation screw tips (infraorbital recess) and the PSI itself (transition zone), which was found in isolated cases, is not associated with chronic sinusitis or impairment of the ostiomeatal complex.

### 5.1. Study Limitations

The presented study has several limitations. In view of the long-term observation period, the number of participants is relatively low (16 dropouts) compared to the cited literature. A selection bias may also exist due to a single-center study and variable trauma complexities. In the presented study, bone remodeling/osseointegration was assessed only qualitatively; quantitative and morphometric analyses would be more robust and appropriate (grayscale). Additionally, confounding parameters are presented in the data set incompletely, as clinical odontogenic factors for the maxillary sinusitis were not recorded. Furthermore, in the presented study, there is a lack of validated patient-reported outcome measures such as SNOT-22. The results and conclusions should be verified in a larger sample in determining its external validity and generalizability to the clinical setting. Despite the limitations, the study may offer valuable contribution to the field by offering insight into the physiology of PNSs.

### 5.2. Clinical Application and Further Research Direction

Successful orbital wall reconstruction beyond accurate bony structures has an impact on ocular function and likewise on paranasal sinus physiology. Therefore, in patients suffering from complex midface injuries, the authors propose a tool to quantitatively assess the paranasal sinuses in the clinical routine. A Lund–Mackay sinusitis stage calculator, web-based and as a mobile application, is available by QxMD from 2019 URL https://qxmd.com/ (lastly accessed on 21 August 2025).

Additionally, for future studies, a validated patient-reported outcome measure, such as the SNOT-22, should be implemented pre-operatively and long-term post-operatively for multivariate regression analysis. This tool may help to quantify changes in clinical symptoms.

Furthermore, the fate of titanium PSIs and the condition of PNSs should also be analyzed through decades, especially in elderly patients. Future research should be directed to the development of slowly resorbable, osteoinductive PSIs, which maintain the advantages of the biocompatible and dimensionally stable titanium.

## Figures and Tables

**Table 1:** Patients and documented parameters according to the PICOST format [26].

Field	Population	Intervention	Control	Outcome	Setting	Time
Craniomaxillofacial surgery	Inpatients who required orbital reconstruction due to trauma	Virtual surgical planning and reconstruction with patient-specific CAD/CAM implants (PSIs)	Contralateral uninjured structures of the midface	Primary outcome measure: status of the paranasal sinuses in relation to the PSI (Lund score [24]). Secondary outcome measures: post-operative (implant-associated) complications, other adverse events	Group of consecutively enrolled patients (February/2019 to May/2024) Department of Oral, Maxillofacial and Plastic Surgery (University Hospital Halle)	Post-operative follow-up period ≥ 6 months to wait for stable wound healing and to record possible complications; the clinical and radiological findings at the last outpatient follow-up were decisive

CAD: Computer-aided design; CAM: computer-aided manufacturing; PSI: patient-specific implant.

**Table 2:** Classification of treatment results for orbital fractures according to Jansen and coworkers [28].

Classification	Diplopia, Enophthalmos, Other Complications or Consequences
Excellent	No diplopia, enophthalmos 0–2 mm, no other complications or consequences
Good	No diplopia, enophthalmos 0–2 mm, other minor complications or consequences
Acceptable	Diplopia outside the field of vision (no compensatory head rotation), enophthalmos 0–2 mm
Poor	Diplopia within the field of vision (no compensatory head rotation), enophthalmos > 2 mm
Failure	Revision due to persistent dysfunctions, diplopia, enophthalmos, other serious complications or consequences

**Table 3:** Cohort of patients with midface trauma involving the orbita.

Patient	Gender	Age	Primary Trauma Diagnosis	Primary Treatment	Year of Surgery	Classification [22]	Access	Duration of Surgery (min)	Ophthalmological Status (Post-Operative, Tensio mmHg)	Complications (Posttraumatic/Post-Operative)
1	F	71	Orbital floor	-	2020	Category II, 1 PSI	Mediopalpebral	60	Visus right 0.8 s.c, left 1.0 s.c	None
2	F	51	Orbital floor	Monitoring	2020	Category III, 1 PSI	Transconjunctival-transcaruncular	105	Visus right 0.2, left 0.8, Tensio right/left normal (10–21 mm Hg)	Lower lid entropion, trichiasis
3	M	48	Orbital floor	Soft tissue reconstruction, removal of foreign bodies	2022	Category III, 1 PSI	Mediopalpebral	64	Visus right 0.6, left 0.6, Tensio right 18, left 14	Initial diplopia; prisms
4	F	49	Orbital floor, open, infraorbital soft tissue laceration	Soft tissue debridement	2023	Category II, 1 PSI	Accidental wounds, subcilliar	107	Visus right 0.6, left 0.8, Tensio right 14, left 12	Lower lid ectropion (result of trauma), preexisting strabismus convergens
5	F	41	Orbital floor, zygomatic bone	Wound care	2020	Category III, 1 PSI	Mediopalpebral	116	Visus right 0.8, left 0.8, Tensio right 18, left 22, swelling	None
6	M	55	Orbital floor, medial orbital wall	-	2020	Category III, 1 PSI	Transconjunctival-transcaruncular	70	-	None
7	M	34	Orbital floor, zygomatic bone	Osteosynthesis upper extremities	2020	Category III, #, 1 PSI	Mediopalpebral	120	Visus right 1.0, left 1.0, Tensio right 12, left 12	None
8	F	71	Orbital floor, infraorbital rim, paranasal buttress comminuted	Osteosynthesis, Infraorbital and paranasal	2023	Category III, #, 1 PSI	Infraorbital	88	Visus right 1.0/left 0.6, Tensio right 15, left 16	Infraorbital dysesthesia
9	F	45	Centrolateral midface (orbital floor, medial orbital wall, NOE complex), frontal base both sides, open, comminuted	Frontobasal coverage, osteosynthesis, ethmoid bone debridement	2019	Category V+, 2 PSIs	Accidental wounds, transconjunctival	295	Visus right 0.3, left 1.0., Tensio r/l normal	Diplopia outside the field of vision, potential opticus lesion
10	M	56	Centrolateral midface both sides, open, comminuted	Soft tissue wound care	2020	Category IV+, ##, 1 PSI	Transconjunctival	213	Visus right 0.8, left 0.8. Tensio r/l 13, Contusio bulbi	Traumatic crooked saddle nose with nasal obstruction, no diplopia
11	F	26	Centrolateral midface (orbital floor, NOE complex, zygomatic bone, zygomatic arch, maxilla), comminuted, (condylar process, right mandible)	Monitoring, soft tissue reconstruction, initial osteosynthesis of midfacial buttresses	2023	Category III, 1 PSI	Mediopalpebral	110 (initial surgery 464)	Visus right 0.8, left 1.0, Tensio right 15, left 15	Diplopia outside the field of vision (upgaze), infraorbital hypoesthesia
12	M	32	Centrolateral midface right, comminuted	Soft tissue reconstruction, eyeball reconstruction, monitoring	2021	Not classifiable (blow-out fracture of all 4 orbital walls, category V+), 3 PSIs	Accidental wounds, combination	360	Visus-, Tensio normal, Hyposphagma right	Amaurosis (questionable lux, blast trauma), soft tissue deficit paranasal and on the nasal wing
13	M	70	Centrolateral midface both sides, frontal base, comminuted	Soft tissue reconstruction, intracranial pressure probe, monitoring	2021	Category III, 1 PSI	Mediopalpebral	187	Visus right 1.0, left 0.4, Tensio right 15, left 12, Contusio bulbi	None
14	M	34	Centrolateral midface (orbital floor, zygomatic bone, zygomatic arch, nasal framework, maxilla) right	Soft tissue reconstruction, tracheotomy, monitoring	2021	Category III, 1 PSI	Accidental wounds, mediopalpebral	80	-	Lesion of the brachial plexus (result of trauma—traffic accident); neurosurgical reconstruction
15	M	28	Centrolateral midface (zygomatic bone, zygomatic arch, maxilla, nasal framework, orbital floor, medial orbital wall) both sides	Soft tissue reconstruction, foreign body removal, monitoring, pre-formed titanium mesh implant for orbital floor and medial orbital wall	2022	Category V+, ##, 2 PSIs	Transconjunctival-transcaruncular with canthotomy	302	Visus right 1.0, left 0.2. Tensio right 15, left 17	Visual acuity 0.25, traumatic mydriasis (iris sphincter tear), scarred lower lid ectropion, left eyebrow ptosis (result of trauma)
16	M	62	Centrolateral midface (zygomatic bone, zygomatic arch, maxilla, nasal framework, orbital floor, orbital roof)	Monitoring, soft tissue reconstruction, initial osteosynthesis of midfacial buttresses	2024	Category V+, ##, 1 PSI	Mediopalpebral	83 (initial surgery 196)	Visus right 0.4, left 0.6. Tensio right 18, left 17	Infraorbital paresthesia and lymphoedema

M: male; F: female; (#): Additional osteosynthesis at the infraorbital rim; (##): Secondary orbital reconstruction; NOE: Naso–orbito–ethmoidal complex.

**Table 4:** Clinical and radiological findings of the last follow-up examination after orbital reconstruction using PSI.

Patient	Follow-Up Interval	Clinical Findings	Radiological Findings				
		Local Complications, Correction	Patent Ostiomeatal Complex	Lund Score (Injured/Uninjured Orbit)	Exposure of Screw Tips	Exposure of the PSI	Bone Apposition at the PSI
1	July 2020 September 2022 (26 months)	Occasional serous rhinorrhea, infraorbital paresthesia	Yes	0/0	Yes	No	Yes
2	October 2020 November 2022 (25 months)	None	Yes	0/0	Yes	No	Yes, partially on the medial wall, dorsally protruding PSI end (4 mm)
3	March 2022 January 2023 (10 months)	Occasional serous rhinorrhea	Yes	0/1	1 screw tip	No	Yes
4	August 2023 February 2024 (6 months)	Lower lid ectopion, “finger flap”	Yes	1/0	No	No	Yes
5	June 2020 October 2022 (28 months)	Infraorbital hyperesthesia	Yes	0/0	No	No	Yes
6	July 2020 November 2022 (28 months)	None	Yes	0/0	No	Minor, ethmoidal, transition zone	Yes
7	August 2020 October 2022 (26 months)	-	Yes	1/0	No	No	Yes
8	October 2023 October 2025 (24 months)	Infraorbital dysesthesia	Yes	0/0	No	No	Yes
9	February 2019 April 2021 (26 months)	Asymmetry, telecanthus on the right, scars in the nasal region	Yes	5/5	No	Ethmoidal	Yes
10	March 2020 January 2022 (22 months)	Anosmia, migraine	Mucosal swelling	3/5	No	Ethmoidal	Yes
11	December 2023 February 2025 (14 months)	Infraorbital hypesthesia	No	6/3	Yes	No	Yes
12	February 2021 June 2022 (16 months)	Amaurosis, blepharophimosis, soft tissue deficit of the nasal wings; forehead flap planed	Yes, additionally neo-infundibulum	2/1	No	Minor, ethmoidal transition zone	Yes
13	June 2021 September 2022 (15 months)	None	Yes	1/0	No	No	Yes
14	July 2021 September 2022 (14 months)	Infraorbital hypoesthesia	No	5/4	No	Covered with mucosa	Minor
15	August 2021 December 2022 (16 months)	Discrete hypoesthesia, corneal scar, traumatic cataract, mydriasis, eyebrow ptosis, antimongoloid eyelid axis position; eyebrow lift, tarsal strip procedure	Yes	0/0	No	No	Yes
16	May 2024 January 2025 (8 months)	None	Yes neo-infundibulum	2/0	No	No	Yes

## Data Availability

The relevant data presented in this study are available in Table 3 and Table 4; others are available on request from the corresponding author due to privacy restrictions.

## Supplementary Materials

The following supporting information can be downloaded at: https://www.mdpi.com/article/10.3390/jcm14207439/s1, Video S1: axial CT examination of patient 13 in Table 3 with complex midface trauma.
